# Triple gallbladder: radiological review

**DOI:** 10.1007/s12328-023-01829-3

**Published:** 2023-08-11

**Authors:** Eliseo Picchi, Paola Leomanni, Vito Bruno Dell’Olio, Noemi Pucci, Francesca Di Giuliano, Valentina Ferrazzoli, Silvia Minosse, Maurizio Rho, Marcello Chiocchi, Francesco Garaci, Roberto Floris

**Affiliations:** 1 Diagnostic Imaging Unit, Department of Biomedicine and Prevention, University of Rome Tor Vergata, Viale Oxford 81, 00133 Rome, Italy; 2 Department of Biomedicine and Prevention, Neuroradiology Unit, University of Rome Tor Vergata, Viale Oxford 81, 00133 Rome, Italy; 3 Department of Surgical Science, University of Rome “Tor Vergata”, 00133 Rome, Italy

**Keywords:** Triple gallbladder, Multiple gallbladders, Triple cholecyst, Multiple cholecyst

## Abstract

Triple gallbladder represents a rare congenital anatomical abnormality that can be a diagnostic challenge in reason to its rarity and consequential difficulties with diagnosis and identification. A systematic review of all published literature between 1958 and 2022 was performed. We identified 20 previous studies that provided 20 cases of triple gallbladder; our case was also included in the analysis, making a total of 21 patients. All patients underwent on diagnostic imaging examinations. After 1985, 9 patients underwent US examination which allowed prompt recognition of triple gallbladder in 2 patients only. CT was performed in 3 patients and allowed the correct diagnosis in a case. In 4 patients, was performed MRCP which allowed the correct diagnosis of triple gallbladder in all patients. Preoperative imaging allows the recognition of triple gallbladder in 9 of 21 patients (43%); in 12 patients (57%) the diagnosis was intraoperative. On patients considered, 16/21 underwent cholecystectomy. In 15 cases, the excised gallbladders were submitted for histopathological characterization with detection of metaplasia of the mucosa in 3 patients, while papillary adenocarcinoma was found in one. Imaging plays a key role in the identification of the anatomical variants of gallbladder, especially triple gallbladder, as modern imaging techniques allow a detailed assessment of the course of the biliary tract for a correct preoperative diagnosis. It is also crucial to be aware of the association between this condition and the metaplasia phenomena with the development of adenocarcinoma, as this may influence the patient’s course of treatment.

## Introduction

Triple gallbladder, also called *vesica fellea triplex*, was first reported by Huber in 1752 during an autopsy [1]; it is an uncommon congenital and often undetected abnormality of the biliary system and, up to now, only 20 cases have been reported in the literature. Therefore, the identification of some congenital gallbladder anomalies, due to their rarity, might represent a significant clinical challenge [2]. As a matter of fact, most of the extrahepatic biliary system abnormalities come to medical attention only when symptoms, that are mainly related to cholelithiasis and cholecystitis, occur.

The aim of this study was to evaluate the cross-sectional imaging findings in patients with triple gallbladder through a literature review as it is associated with an increased rate of gallbladder metaplasia, dysplasia, and adenocarcinoma: for these reasons, the prompt identification of this anatomical variant is crucial.

## Case report

A 56-year-old female patient reported acute abdominal pain in the right hypochondrium and mild nausea after a large meal; the pain spontaneously regressed in about 24 h after onset. The patient had no clinical history of either biliary colic or biliary tract disease, never had any kind of abdominal surgery; no comorbidity were reported.

Blood tests performed 7 days after the acute episode revealed an increase in total (2.56 mg/dl) and indirect bilirubin (0.65 mg/dl); serum tumor markers (CEA and CA 19.9) were negative.

**Fig. 1:**
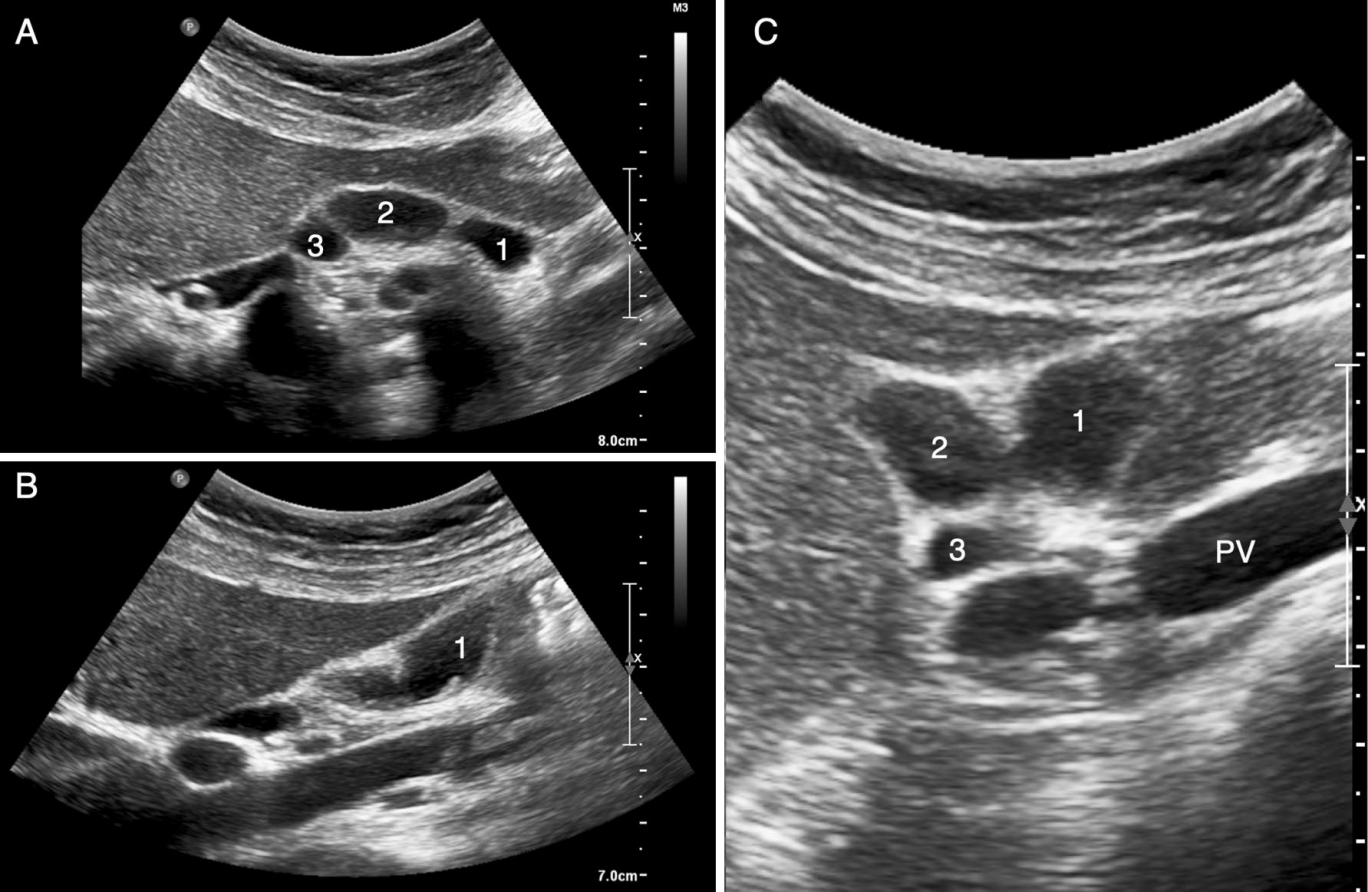
Ultrasound examination showed three different gallbladders (numbers 1, 2, 3 in panels **A** and **C**) in the cholecystic fossa. To note the diffuse thickened, irregular and hyperechogenic wall of the most anterior gallbladder (panel **B**, number 1). *PV* portal vein

The patient underwent ultrasound (US) examination, performed with a high-definition multiband convex probe. The US showed three closely adjacent gallbladders in the cholecystic fossa. One of the gallbladders was hypo-distended, with diffuse thickened and irregular hyperechogenic walls with focal thickening at the fundus (Fig. 1). No dilation of intra- and extrahepatic biliary system was detected. However, ultrasonographic examination did not allow a detailed study of the anatomy of the cystic ducts and their relationship with the extrahepatic biliary tree, neither of all the three gallbladders: for these reasons, magnetic resonance cholangiopancreatography (MRCP) was performed.

The MR examination was performed using a high-field magnetic scanner (1.5 Tesla) with axial T2-weighted (w) and T2w-SPAIR sequences, T1w-DUAL FFE sequence, diffusion weighted imaging and completed with MRCP study obtained with radial, volumetric and gradient and spin echo (GRaSE) sequences.

**Fig. 2:**
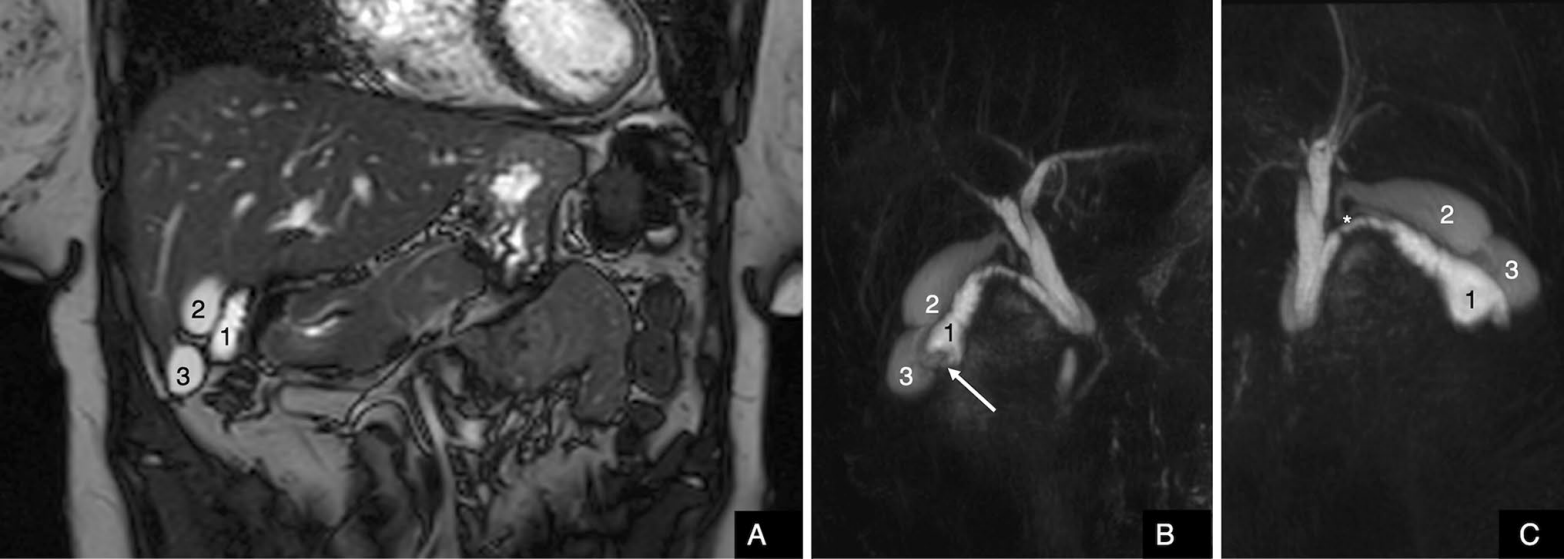
**A** Coronal balanced turbo-field-echo (BTFE) sequence showed three different gallbladder one of which with irregular wall. **B** Anterior view of 3D-MIP magnetic resonance cholangiopancreatography (MRCP) with gradient and spin echo (GRASE) technique; to note the anterior gallbladder with irregular wall, fundal thickness (arrow) and higher T2 signal than two others. **C** Posterior view of 3D-MIP magnetic resonance cholangiopancreatography (MRCP) with gradient and spin echo (GRASE) technique; to note the confluence of the two posterior cystic ducts into a single duct (*)

**Fig. 3:**
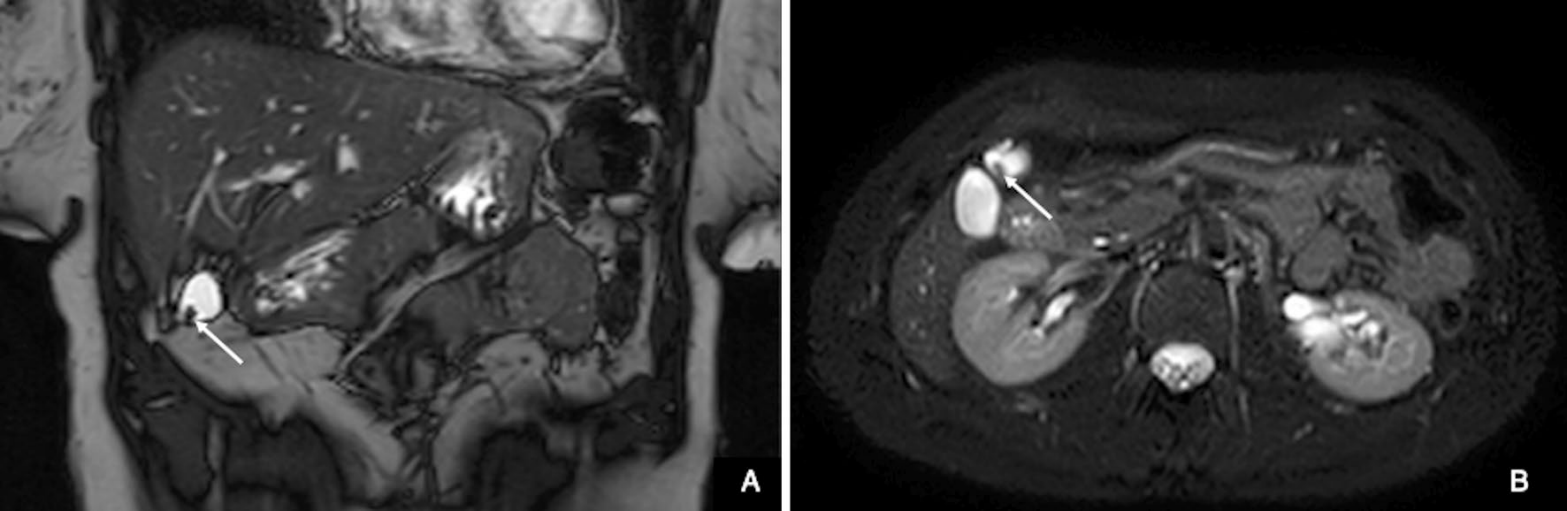
**A** Coronal balanced turbo-field-echo (BTFE) and **B** transverse T2-Spectral Adiabatic Inversion Recovery (SPAIR) sequence: to note the gallbladder with nodular thickness at fundus (white arrow)

**Fig. 4:**
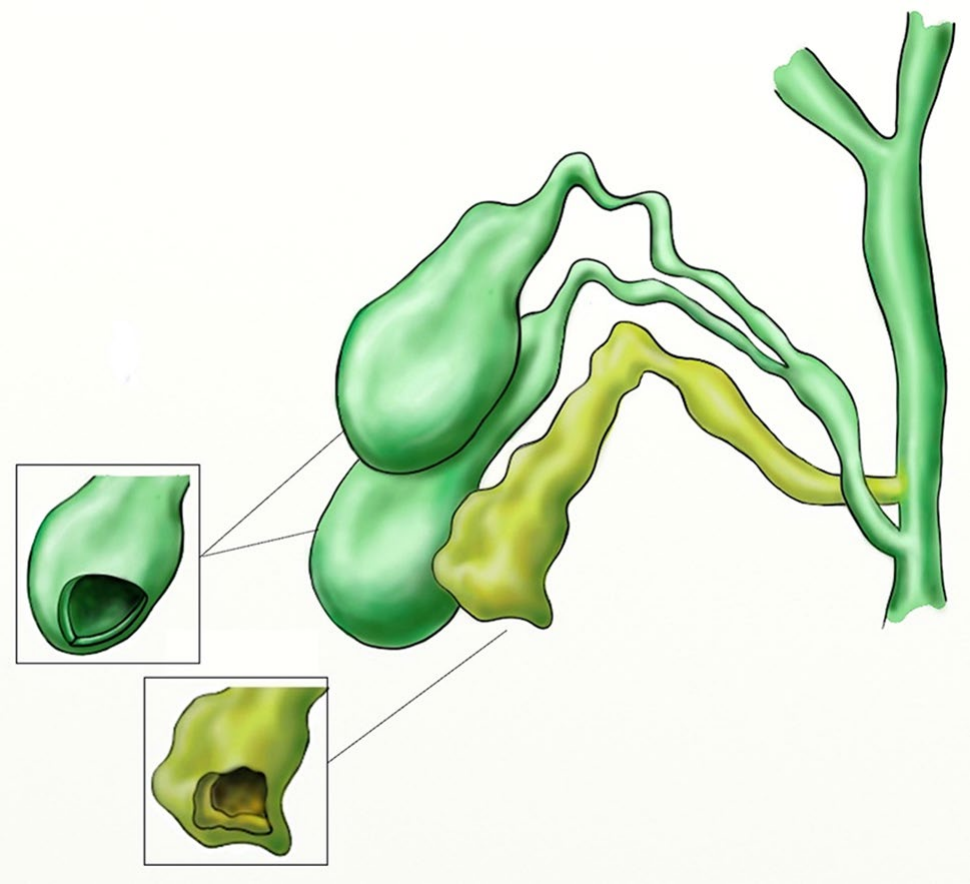
Illustration on coronal plane of our case showing the Y + H triple gallbladder morphology following the Harlaftis classification and the focal fundus wall thickness

**Fig. 5:**
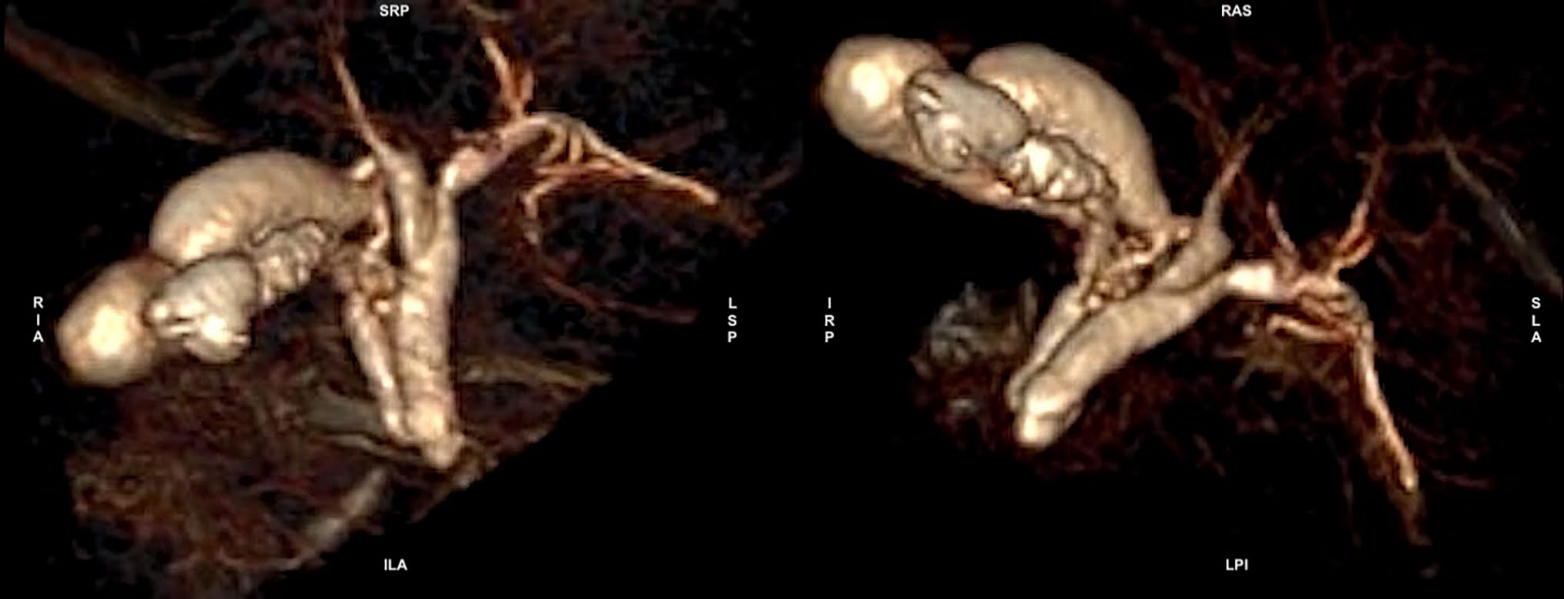
3D volume rendering reconstruction showing three different gallbladders one of which with irregular wall. *RIA* right-infero-anterior; *SRP* supero-right-posterior, *ILA* infero-left-anterior, *LSP* left-supero-posterior; IRP infero-right-posterior, *RAS* right-antero-superior, *SLA* supero-left-anterior, *LPI* left-postero-inferior

**Fig. 6:**
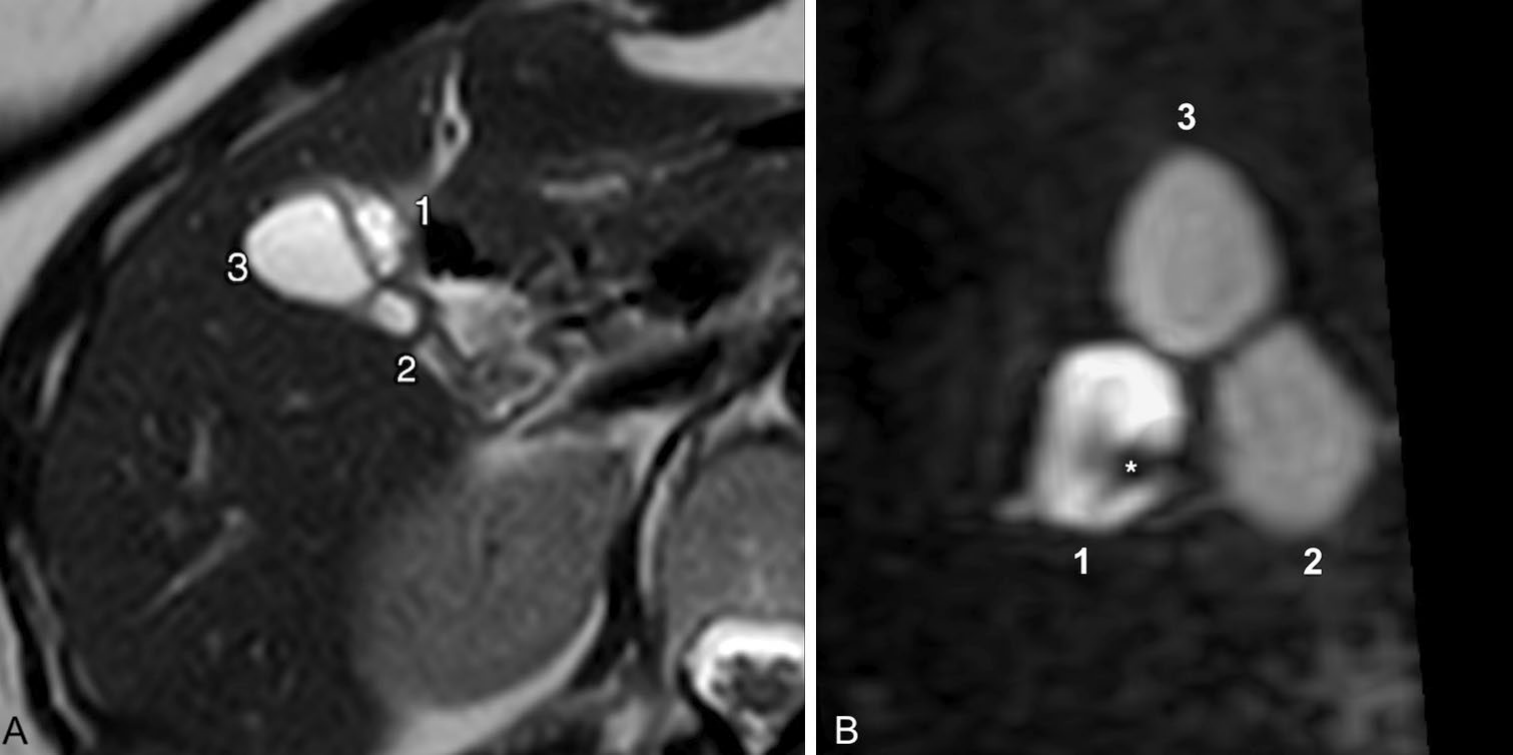
**A** Transverse T2 showing three distinct gallbladders (numbers 1, 2 and 3) [123]. **B** Infero-lateral view of 3D-MIP magnetic resonance cholangiopancreatography (MRCP) with gradient and spin echo (GRASE) technique; to note the fundus of the three distinct gallbladders [123], the anterior with fundal thickness (*) and higher T2 signal than two others

**Fig. 7:**
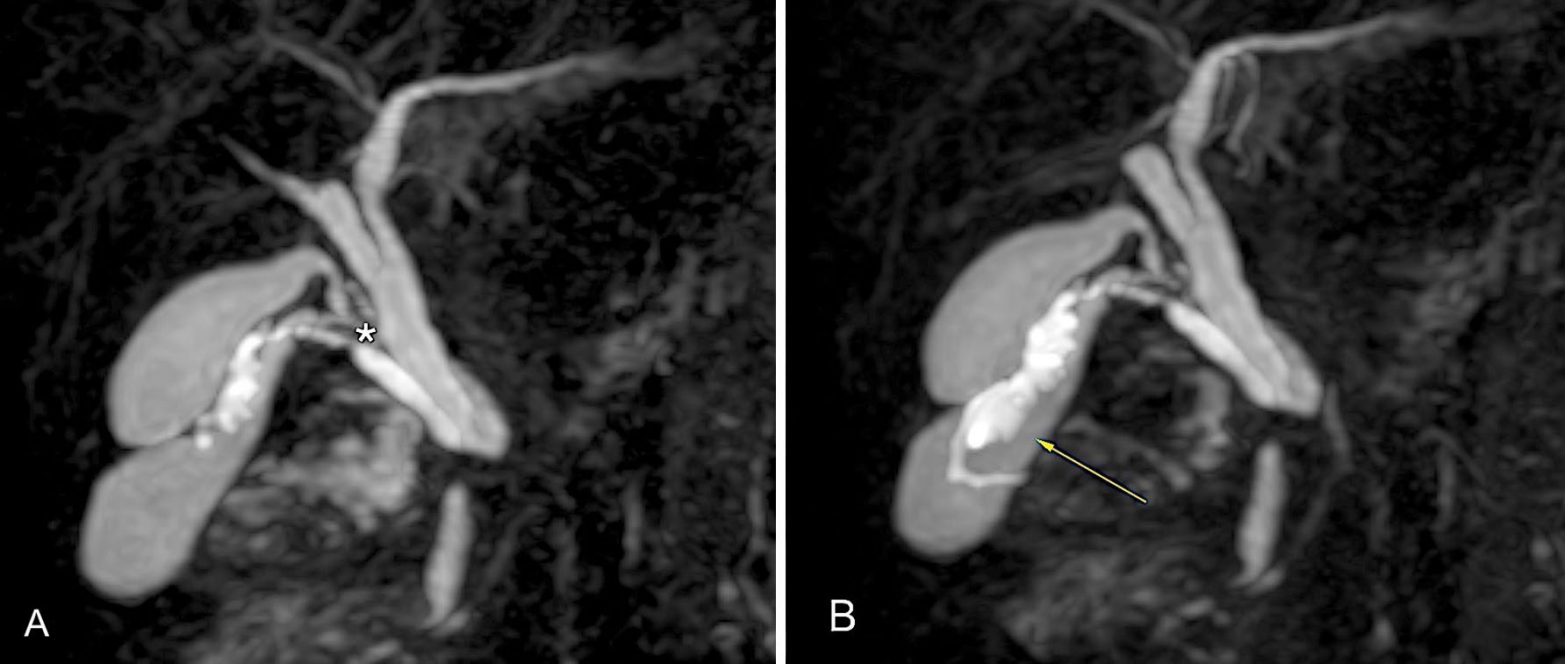
Anterior view of 3D-MIP magnetic resonance cholangiopancreatography (MRCP) with gradient and spin echo (GRASE) technique; to note the anterior gallbladder with irregular wall, fundal thickness (arrow) and higher T2 signal than two others and the confluence of the two posterior cystic ducts into a single duct (*)

The study confirmed the presence of three distinct gallbladders located in the cystic fossa (Figs. 2, 3, 4, 5, 6, 7):

- the most anterior gallbladder had a more hyperintense endoluminal T2 signal and was characterized by irregular walls and multiple parietal thickenings with polypoid-like appearance. The largest wall thickening was at the fundus (6 mm). The cystic duct arising from this gallbladder showed mild ectasia (5 mm) and confluence on the posterior wall of the middle third of the choledochal duct (Figs. 6, 7).

- the two posterior gallbladders had regular walls and their respective cystic ducts converged into a single duct which joined the choledochal duct at the level of the postero-medial wall, just below the insertion of the cystic duct of the anterior gallbladder (Figs. 2, 3, 4, 5, 6, 7).

According to the Harlaftis classification [3], the presented triple gallbladders had Y + H morphology (Fig. 4).

MRCP showed a focal filling defect in the intrapancreatic choledochal duct immediately downstream the confluence of the cystic ducts, with ectasia of the upstream segment (7 mm); the pre-papillary segment of the choledochal duct showed a maximum diameter of 5 mm with no evidence of endoluminal signal abnormalities (Figs. 2, 7).

Patient underwent to surgical examination during which she was informed about the clinical risks associated with this rare condition and even though laparoscopic cholecystectomy was proposed to remove the triple gallbladder, and the patient refused surgery.

## Methods

We performed a literature search for case description of triple gallbladder in humans published up to September 2022, using the keywords “triple cholecyst”, “triple gallbladder”, “multiple cholecysts” and “multiple gallbladder” without additional filters. Eligible articles were fully reviewed for compliance with the research objectives and adequacy of data. Studies reporting a clear description of the triple gallbladder found during radiological examinations or following cholecystectomy surgery were considered eligible. In addition, we included the present case report. Radiological features and clinical data were collected and analyzed by descriptive statistics.

The study was conducted according to local ethical standards and Helsinki Declaration’s principles.

## Results

From the literature review, we identified 20 previous studies that provided 20 cases of triple gallbladder; our case of triple gallbladder was also included in the analysis, making a total of 21 patients with triple gallbladder. The clinical and anamnestic characteristics of the patients, radiological examinations, laboratory tests, surgeries performed, and histopathological findings are summarized in Table 1.

**Table 1:** The table, sorted by data, shows a comprehensive review of the demographical, symptoms, blood test values, radiological and histopathological findings of patients with triple gallbladder

Authors	Age/gender	Symptoms	Blood tests	Radiological findings	Type	Surgery	Histopathological findings
Our report	56 F	-Abdominal pain	↑ Total bilirubin (2.56 mg/dL) bilirubin (0.56 mg/dL)	US revealed: three gallbladders in the cholecystic fossa MRCP revealed: confirmed the presence of three gallbladders and evaluate the anatomy of the biliary tract	II	NP	NP
Chen X, Yi B [12]	5 F	-Abdominal pain	↑ ALT (465 U/L)	CT and MRCP revealed: a) Triple gallbladder b) Three cystic ducts c) A shared common bile duct d) Choledocholithiasis e) Dilated pancreatic duct	I	Laparoscopic cholecystectomy	-Chronic cholecystitis -Wall edema -Gallbladder adhesion
Ott L et al [8]	2 F	-Abdominal pain -Emesis	↑ ALT (359 U/L) ↑ AST (146 U/L) ↑ ALP (549 U/L) ↑ GGT (515 U/L)	US revealed: a) Intrahepatic and extrahepatic biliary dilation b) Tubular cystic structure anterior to the gallbladder that communicated with the common bile duct MRCP revealed: a) Mild dilation of the central intrahepatic biliary ducts b) Two tubular cystic structures superior to the gallbladder communicating with the common bile duct	I	Laparoscopic cholecystectomy	-Chronic cholecystitis -Gastric metaplasia with oxyntic cells
Copeland-Halperin LR et al. [26]	30 M	-Abdominal pain -Icterus -Fever -History of cholelithiasis and choledocholithiasis	↑ Total bilirubin (12.2 mg/dL) ↑ Direct bilirubin (7.9 mg/dL) ↑ ALP (218 U/L) ↑ ALT (105 U/L) ↑ AST (38 U/L)	US revealed: a) Cholelithiasis b) Gallbladder sludge b) Wall thickening CT revealed: a) Mass in the gallbladder fossa	I	Cholecystectomy Intraoperative diagnosis of triple gallbladder	-Gallbladder hydrops -Necrotic and hemorrhagic walls
Khadim MT et al. [1	30F	-Abdominal pain -Nausea	Leukocytosis ↑ ALP	US revealed: a) Duplicated gallbladder b) Thickened wall c) Multiple gallstones	II	Open cholecystectomy Intraoperative diagnosis of triple gallbladder	-Fibrosis -Mononuclear cell infiltrate -Multiple gallstones
Alicioglu B [16]	54 F	-Abdominal pain -Nausea -Dyspepsia	Leukocytosis ↑ ALT (167 U/L)	US and CT revealed: a) Duplicated gallbladder, with thickened wall and considerable sludge in one gallbladder b) Tortuous cystic in the last gallbladder ERCP: two cystic ducts, normal common hepatic duct	I	Cholecystectomy	- Ulcerated active chronic cholecystitis
Barnes S el al [7]	15 F	-Epigastric pain -Emesis	Within the limits	US revealed: 3 fluid-filled structures in the GB fossa. GB triplication was suggested MRCP revealed: 3 separate GB. Two of the cystic ducts could be visualized becoming a common cystic duct. This common cystic duct was shown to enter a normal-looking CBD	III	Laparoscopic cholecystectomy Each of the GBs had its own small cystic duct, and all converged into a larger cystic duct, which entered a single CBD	-Non-inflamed triple GB
Mottin CC et al. [27]	36 F	-Abdominal pain	Within the limits	US revealed: gallbladder stones	I	Cholecystectomy: three independent gallbladders were identified, each one communicating with the common bile duct through its own cystic duct	NP
Schroeder C [28]	38 M	-Biliary colic -Fever	-Leukocytosis (14,000 mm^3^)	US revealed: two gallbladders with stones	I	Laparoscopic cholecystectomy: the diagnosis was intraoperative	-Chronic inflammatory disease; -Lithiasis
Nanthakumaran S et al. [6]	50 F	-Biliary colic -Clinical history of choledocholithiasis	NA	US and ERCP revealed: a) lithiasis b) no abnormality of biliary tract	I	Laparoscopic cholecystectomy: intraoperative diagnosis	-Lithiasis -Chronic cholecystitis
Gruk M et al. [29]	29 M	-Abdominal pain	No abnormalities	Cholecystography: non-diagnostic examination	I	Cholecystectomy	-Lithiasis
Foster DR [15]	46 M	-Abdominal pain	NA	Cholecystography and CT revealed: a) three separate gallbladders b) there were calculi present in one of the gallbladders c) single cystic duct draining into the common duct d) the cystic ducts of the other two gall bladders fused prior to insertion into the common bile duct	II	NP	NP
Kurzweg FT el al [5]	34 M	-Hematemesis	NA	Cholecystography revealed: triple gallbladder with a common cystic duct	III	NP	NP
Roeder WJ et al. [11]	36 M	-Abdominal pain -Icterus -Fever -History of fever	-Leukocytosis (11,500/mm^3^)	Oral cholecystography revealed: poorly functioning gallbladder containing a solitary round calcific density measuring 3 cm	I	Open cholecystectomy: intraoperative diagnosis The “third” gallbladder was not removed	Examination of the “first” gallbladder showed the mucosal surface was hemorrhagic -Cholelithiasis and subacute and chronic cholecystitis -Papillary adenocarcinoma
Aroca Ruiz-Funes J.M. et al. [20]	43 F	-Chronic abdominal pain -Nausea -Fever	↑ ALP ↑ GGT ↑ Direct bilirubin	Cholecystography revealed: triplication of the gallbladder	III	Cholecystectomy	-Acute cholecystitis
Ross RJ & Sachs MD [30]	51 M	-Abdominal pain	NA	Cholecystography revealed: triplication of the gallbladder; the largest and independent gallbladder seemed to empty directly into a large cystic duct. Two smaller gallbladders near the infundibulum appeared to join the common duct in a cluster close together	II	NP	NP
Kelly A [9]	12 M	-Biliary colic -Emesis	NA	Cholecystography revealed: two gallbladders each with a cystic duct. These ducts unite just prior to entering the common duct	II	Cholecystectomy: the diagnosis was intraoperative	In the two gallbladders, which have a common cystic duct, there was chronic inflammation -Lithiasis -Duodenal metaplasia
Hause WA [31]	69 F	-Abdominal pain -Nausea -Emesis	NA	Cholecystography revealed: numerous calculi	I	Cholecystectomy: the diagnosis was intraoperative	Dissection revealed there were three separate chambers and a separate cystic duct -The mucosa was coarsened and irregular and the wall thick and tough -Lithiasis -Chronic inflammation
Skielboe B [32]	54 F	-Abdominal pain -History of cholelithiasis	NA	Cholecystography revealed: duplicated gallbladder	I	Open cholecystectomy; the diagnosis was intraoperative	-Edema -Lymphocytic cellular infiltration
Casey EW [10]	12 M	-Abdominal pain -Chronic dyspepsia	NA	Cholecystography revealed: two gallbladders, each with a separate cystic duct, the ducts apparently uniting just before they entered the common bile duct	II	Cholecystectomy: intraoperative diagnosis	-Lithiasis -Duodenal metaplasia
Boni R [4]	32 F	-Abdominal pain	Within the limits	Cholecystography revealed: three gallbladders; lithiasis	NA	NP	NP

*ALP* alkaline phosphatase, *ALT* alanine transaminase, *AST* aspartate transaminase, *CT* computed tomography, *ERCP* endoscopic retrograde cholangiopancreatography, *F* female, *GGT* Gamma-glutamyl Transferase *M* male, *MRCP* magnetic resonance cholangiopancreatography, *NA* not available, *NP* not performed; *US* ultrasound

First case of triple gallbladder was reported by Boni in 1958 [4]. The triple gallbladder is more common in women (12 women and 9 men), with a female/male ratio of 1.33; the mean age at diagnosis was 34 years, specifically 36 years in women and 32 years in men.

The clinical manifestations of triple gallbladder are related to non-specific gastrointestinal symptoms: most patients (20/21 patients—95%) had at least one episode of abdominal pain before diagnosis. The abdominal pain manifested either as a single episode of biliary colic or as repeated self-limited episodes occurring over a variable period of weeks, months or even years.

Only in one patient the diagnosis of triple gallbladder occurred without abdominal pain, and it was found incidentally through radiological examinations performed following an episode of hematemesis [5].

In addition to abdominal pain, 9/21 (43%) patients presented with nausea and/or vomiting, 4/21 (19%) patients presented fever, and 3/21 (14%) had a clinical history of cholelithiasis or choledocholithiasis.

In 13 patients, blood chemistry tests were performed after the onset of symptoms; in particular, we evaluated indices of liver function (aspartate aminotransferase-AST, alanine aminotransferase-ALT, total bilirubin), cholestasis (Gamma-glutamyl Transferase-GGT, Alkaline Phosphatase-ALP, direct bilirubin) and leukocytosis as the main inflammatory index. Among these 13 patients, 4 had normal blood tests; in the remaining 9 patients, cholestasis and leukocytosis indices were abnormal. 6 patients (46%) had at least one increased cholestasis index value and 4 patients (31%) had leukocytosis. An increase in liver function indices was detected in only in 3 patients (23%).

All patients underwent further diagnostic investigation by imaging examinations. Specifically, all the 11 patients reported between 1958 and 1985 underwent cholangiography: this exam allowed the recognition of the triple gallbladder in 5 cases while in 3 cases revealed two gallbladders and in the last 3 cases it was not diagnostic. However, a correct depiction of the extrahepatic biliary anatomy was achieved in 3 patients only.

In the cases published after 1985, 9 patients underwent US as the first-level imaging examination: US allowed prompt recognition of triple gallbladder in 2 patients only, while in 4 patients, it was wrongly identified a duplicate gallbladder. Moreover, US rarely allows correct biliary tree anatomy depiction.

CT was performed only in 3 patients and allowed the correct diagnosis of triple gallbladder in a case.

In 4 patients, the study of the biliary tract was performed by MRCP which allowed the correct diagnosis of triple gallbladder in all patients, even though in a single patient, it gave incomplete information about the anatomy of the biliary tree.

ERCP was performed in only in 2 patients, and in both cases, it was non-diagnostic.

Considering the available data, preoperative imaging allows the recognition of triple gallbladder in 9 of 21 patients (43%) only; in the remaining 12 patients (57%), the diagnosis was intraoperative.

All reported cases of triple gallbladder were classified according to the anatomy of the cystic ducts [3], specifically the most frequently encountered anatomical variant was type I (11/20 patients, 55%) followed by type II (6/20 patients, 30%) and type III (3/20 patient 15%).

Only for a patient, the anatomy of cystic ducts was not defined and for this reason, it has not been reported [6].

Of the total 21 patients considered, 16 underwent to cholecystectomy. In 15 cases, the surgical samples of the excised gallbladder were submitted for histopathological characterization and the most common histologic finding was chronic cholecystitis (11/15 patients, 73%). In 2 patients, the walls of the excised gallbladder had necrotic and hemorrhagic features. Only a patient had no histological changes of gallbladder [7].

Metaplasia of the gallbladder mucosa was found in 3 patients [8–10], of which 2 had duodenal mucosa [9, 10] and one had gastric mucosa and ossicular cells [8].

Dysplastic mucosa with papillary adenocarcinoma was found in one patient [11]. The presence of endoluminal lithiasis was found in 9 patients (43%).

## Discussion

During the 4th week of gestation, hepatobiliary and pancreatic organogenesis begins from the endodermal bud originating from the anterior intestine [12]; specifically, its upper segment gives rise to the liver and intrahepatic bile ducts and its lower segment to the gallbladder, cystic duct, and common bile duct [13]. Between the 4th and 5th week of gestation, the gallbladder primordium begins to form, which will give rise to the gallbladder and cystic duct: interruption or alteration of the development process at this stage may result in malformations of the gallbladder and extrahepatic bile ducts [12]. In 1926, Boyden hypothesized that multiple gallbladders represented an outgrowth of a secondary vesicle from a portion of the bile duct system after the formation of the definitive gallbladder [14]. In the human embryo, this small cellular opening, called the rudimentary bile duct, usually regresses, and disappears [15]; however, the failure of the rudimentary bile ducts to regress can lead to the formation of extroversions along the biliary system, and when it occurs along the extrahepatic pathways, it can result in the development of accessory gallbladders [8].

Depending on the location of these accessory sacs, different anatomical relationships between gallbladders, cystic ducts and common hepatic duct will take place: if the buds that give rise to the accessory gallbladders originate from the common hepatic duct, the gallbladders will have independent and separate cystic ducts; on the other hand, if the sacs originate from a cystic duct there will be gallbladders with a common cystic duct draining into the common hepatic duct [1].

In 1977, Harlaftis et al. proposed an anatomical classification of multiple gallbladder based on the embryogenetic mechanism [3]:

-type I consists of a double gallbladder with more or less separate cystic ducts jointly discharging into the common hepatic duct;
-type II consists of a double gallbladder with two independent cystic ducts discharging separately into the biliary tree; in this case, the accessory gallbladder may reach the common hepatic duct (ductular type) or an intrahepatic bile duct (trabecular type);
-type III includes gallbladder with anatomical anomalies that do not fit the previous two groups.

Based on the number and morphology of the cystic ducts, three different types of triple gallbladder have been identified by Alicioglu [16]:

- the first type is characterized by the presence of three gallbladders each with its own independent cystic duct hat drains separately into the bile duct;
- the second type is characterized by the presence of two gallbladders with a common cystic duct draining into the common bile duct and a third gallbladder with independent cystic duct;
- the third type is characterized by the presence of three gallbladders sharing a single cystic duct.

From our analysis, the most common type of triple gallbladder was type I (55%) followed by type II (30%) and finally type III, the most rarely encountered variant (15%).

Multiple gallbladder is a very uncommon condition and, usually, its clinical presentation is non-specific [2, 16]. Colic abdominal pain is the most commonly reported symptom and it is related to cholecystitis and cholelithiasis [13]. Accessory gallbladders may be found incidentally in asymptomatic patients during a routine radiological examination [16].

Preoperative diagnosis of the triple gallbladder condition is crucial, as it allows proper planning of surgery and consequently also a reduction of possible intra- and post-operative complications.

Until 1979, preoperative imaging of the biliary tract was performed by oral or intravenous cholecystography, fat meal studies and conventional tomography. Currently, preoperative imaging uses high-resolution techniques such as US, spiral or multilayer CT, MRCP, and ERCP.

The most frequently performed first-level examination in the case of patients with colic symptoms related to gallbladder is US, as it is highly available and low cost [16]. However, US is an operator-dependent imaging modality and has a low sensitivity (65%), so this anatomical variation could be missed or misdiagnosed [2, 13].

Both US and CT are able to assess the anatomical features of the gallbladder such as number, wall thickness and presence of endoluminal lithiasis, although they may not depict the exact anatomy of the biliary tree [16].

Therefore, further investigations are recommended. MRCP allows a non-invasive study of the anatomy of the biliary system and its abnormalities, accurately describing the number, the position of cystic ducts and their relationship to the common bile duct [13, 17].

ERCP allows detailed assessment of the anatomy of the biliary system, but it is an invasive method that requires general anesthesia, exposes patients to radiation and it could be associated with the development of serious complications [2].

To now, MRCP has proven to be much more sensitive than ERCP, with a sensitivity of about 97% in identifying multiple gallbladder condition [13].

Total cholecystectomy with removal of all gallbladders is the appropriate treatment for symptomatic gallbladder triplication. The literature suggests that benefits from removing of all gallbladders are crucial once surgery is decided. Several studies have been reported describing the need for a second operation because symptoms were not relieved after the first cholecystectomy [18].

Prophylactic surgery is not recommended for incidentally discovered asymptomatic triplication gallbladder, but radiological follow-up is fundamental to early diagnose a possible development of biliary dysplasia or cancer.

Laparoscopic cholecystectomy is performed in most cases, but open approach is preferred in case of the high insertion of the cystic ducts, previous abdominal open surgery, and poor experience in laparoscopic surgery [19].

Preoperative imaging study of anatomical variants is essential for a safe surgery. The surgeon should know, preoperatively, the shape and location of gallbladders, abnormalities of the cystic artery and cystic ducts, to avoid important surgical complications, such as iatrogenic bile duct injuries and hepatic vascular injuries.

In most cases, patients with symptomatic multiple gallbladders underwent cholecystectomy.

Histopathological examination of the 15 patients who underwent surgery showed chronic cholecystitis in 9/15 patients (60%) and a cholelithiasis in 12/15 patients (80%). Only one case showed a condition of acute cholecystitis at the histopathological evaluation [20].

Moreover, the abnormal anatomy of the extrahepatic biliary outflow tracts can lead to biliary stasis and thus a predisposition to the formation of endoluminal lithiasis giving rise to a condition of chronic cholecystitis, which is a major risk factors for the occurrence of epithelial metaplasia and the development of gallbladder carcinoma [21, 22].

In fact, consistent with the literature reports [13], the triple gallbladder condition was associated with duodenal metaplasia in 2/15 cases (13%) [9, 10], with gastric metaplasia in 1/15 case (6.5%) [8] and with papillary adenocarcinoma in 1/15 case (6.5%) [11].

In our analysis, intestinal metaplasia was found in 2 cases [9, 10], both associated with a chronic cholecystitis condition, and in one case [9], associated with endoluminal lithiasis.

It is, therefore, very important to diagnose chronic cholecystitis in those anomalies as early diagnosis allows early intervention and prevents tumor development.

This represents a very important risk factor because although it is a very common pathological condition, the incidence of finding dysplasia in gallbladders removed for stones is 13.5%, and of these 3.5% have carcinoma in situ [23].

This condition, depending on both genetic and acquired risk factors [23], can be driven by a condition of gallbladder hypomotility and bile stasis [24].

Intestinal metaplasia represents a frequent finding in almost the 90% of gallbladders with chronic inflammation; this condition has a close relationship with gallstones and wall inflammation which brings to a local chronic response that drives to evolution in focal dysplasia and evolution in gallbladder adenocarcinoma in 3.5% of cases [25].

## Conclusion

Surgeons and radiologists should be aware of the existence of the triple gallbladder condition and the most appropriate imaging methods for proper evaluation of this condition.

Imaging plays a key role in the identification of the anatomical variants of gallbladder, especially triple gallbladder, as modern imaging techniques allow a detailed assessment of the course of the biliary tract for a correct preoperative diagnosis. This allows the surgical procedure to be planned appropriately to reduce intraoperative risk and patient morbidity.

It is also crucial to be aware of the association between this condition and the phenomena of gastric and duodenal metaplasia and with the development of adenocarcinoma, as this may influence the patient’s course of treatment.
